# Evaluation of the Nutritional Impact of Baobab Leaves (*Adansonia digitata* L.) as a Dietary Intervention to Combat Nutrient Deficiencies and Poverty-Related Health Problems

**DOI:** 10.3390/nu16244340

**Published:** 2024-12-16

**Authors:** Abdelhakam Esmaeil Mohamed Ahmed, Massimo Mozzon, Abdaljbbar B. A. Dawod, Eltayeb Omaima Awad Mustafa, Shaikh Ayaz Mukarram, Tahra ElObeid, Elshafia Ali Hamid Mohammed, Béla Kovács

**Affiliations:** 1 Faculty of Agriculture, Food Sciences and Environmental Management, Institute of Food Science, University of Debrecen, Böszörményi str. 138, 4032 Debrecen, Hungary; ayaz.shaikh@agr.unideb.hu (S.A.M.); kovacsb@agr.unideb.hu (B.K.); 2 Doctoral School of Nutrition and Food Sciences, University of Debrecen, Böszörményi út 138, 4032 Debrecen, Hungary; 3 Faculty of Forestry, University of Khartoum, Khartoum North 13314, Sudan; dawod.abdaljbbar@inf.unideb.hu; 4 Department of Agricultural, Food and Environmental Sciences, Marche Polytechnic University, Via Brecce Bianche, 60131 Ancona, Italy; m.mozzon@staff.univpm.it; 5 Doctoral School of Informatics, University of Debrecen, 26 Kassai Road, 4028 Debrecen, Hungary; 6 Sudan Medical Specialization Board, Federal Ministry of Health, Khartoum 11115, Sudan; omaima.awad@med.unideb.hu; 7 Department of Public Health and Epidemiology, Faculty of Medicine, University of Debrecen, 4028 Debrecen, Hungary; 8 Doctoral School of Health Sciences, University of Debrecen, 4028 Debrecen, Hungary; 9 World Food Forum, 00100 Rome, Italy; 10 Department of Nutrition Sciences, College of Health Sciences, QU Health, Qatar University, Doha P.O. Box 2713, Qatar; tahra.e@qu.edu.qa; 11 Agricultural Research Corporation, Integrated Pest Management Reseach Center, Wadmadani 21111, Sudan; elshafia.arc@gmail.com

**Keywords:** health, food insecurity, dietary intervention, baobab leaves

## Abstract

Background/Objectives: Baobab (*Adansonia digitate* L.) is an underutilized species and edible parts (fruits, leaves and seeds) contribute to food security and human health in tropical areas. Although the fruits have attracted greater research interest and have recently been approved for consumption in EU countries, the leaves are traditionally consumed but they have yet to be studied from an interventional perspective. The aim of this study was to propose a protocol for a dietary intervention using baobab leaves (BLs) to achieve the recommended reference values for proteins and minerals (K, Ca, Mg, Na, Fe, Mn) for different target groups of the Sudanese population. Methods: Dry matter, crude fat, protein and ash content, mineral content (Na, Mg, K, Ca, Fe, Mn), total phenolic, and flavonoid compounds were determined in BLs from six different areas. To assess the health and nutrition status in Sudan, time-series data (2013–2023) from the DataBank Health Nutrition and Population Statistics database were used. The reference values for nutrients recommended by the European Food Safety Authority were used to estimate the amount of baobab leaf intake (BLI, g/day). Results: For each nutrient, the study area with the lowest amount of BLs to be consumed is recommended. Leaves from the area of El Gari (BN3) 18.312 g/day and 30.712 g/day are recommended for K and Ca, which are particularly beneficial for children aged 1–3 years and lactating women. Leaves from Kor Tagat (KR1) are suitable for sodium intake, requiring approximately 13–23 g/day across all age groups. Leaves from Kazgil (KR2) (46–81 g/day), (35–66 g/day), (0.48–0.68 g/day), and (4–6 g/day) are optimal for fulfilling the daily requirements of magnesium, iron, manganese, and protein in this order. Conclusions: The systematic inclusion of BLs in the diet can positively support the nutritional status of various demographics. Moreover, the findings of this study demonstrated the foundation for public health and nutritional policy-makers on how they will tackle malnutrition and food insecurity worldwide by incorporating naturally available diets and nutritious alternatives. Recommendation: Further research should focus on assessing the nutritional composition factors that could affect the absorption of nutrients such as phytates and oxalates and investigating the in vitro bioavailability of the elements.

## 1. Introduction

Food insecurity has become a public concern as it affects an extensive range of people worldwide. It is considered food scarcity, the nonexistence of a vital diet, and poor global food. The association between food insecurity and micronutrient deficit needs more effort to extend and deliberate the correlation. Moreover, food insecurity is related to a nutrient deficiency of 89% [1,2]. As endorsed by the 2030 agenda for sustainable development is a need to address food insecurity, hunger, and malnutrition internationally, empathetic these complications consent to the public health policymakers and nutritionists accomplished of contributing to solving the existing issues [3]. Regionally, Sub-Saharan African countries have attracted the most attention in terms of food security including Sudan [4]. According to the integrated food security phase classification (IPC), Sudan showed a high rate of food insecurity, which is considered a critical manner [5]. Furthermore, low health expenditure and prevalent poverty aggravate nutritional deficiencies, mostly among rural populations locally, and internationally [6].

Therefore, millions of people worldwide are affected by nutritional deficiencies, which has led to various public health problems [7]. Tropical areas, including Sudan, are affected by widespread diseases such as anemia, which is positively correlated with inadequate dietary iron intake [8]. In this context, more than 20.3 million Sudanese, about 42% of the total population, face food insecurity due to the ongoing conflict and suffer from malnutrition, especially among children [9,10]. Moreover, Sudanese women often suffer from a lack of essential microelements such as iron during the first trimester of pregnancy [11].

The most important factor influencing human health is the adequate supply of nutrients in the diet [12]. In particular, macro elements, such as calcium, are crucial for developing the immune system. A drop in Ca ion levels leads to immunodeficiency diseases [13]. In addition, insufficient Ca intake leads to a negative Ca balance in the human body, which can lead to osteoporosis and parathyroid hyperplasia [14]. The metabolic reactions of the human body depend mainly on magnesium levels, and 73.8% of women worldwide are affected by Mg deficiency, especially in the fertile age [15]. There is a significant association between the level of Mg deficiency and sleep quality in older adults; therefore, adequate Mg intake in the daily diet can mitigate this problem [16]. In addition, connective tissue diseases are associated with Mg deficiency [17].

In light of global food shortages and health problems, a systematic approach is needed to deal with people’s lives [18]. A diet rich in a variety of fruits and vegetables is one of the best practices for overall human health and well-being [19]. Baobab (*Adansonia digitata* L.) is an underutilized species with important edible parts that play a crucial role in food security to protect people from malnutrition. Traditionally, baobab leaves powder has been added to the sauce prepared from different ingredients such as onion, tomato, salt, and dried fish, then baobab fruit pulp was added to the porridge made from maze, both recipes were formulated to be consumed by children of age 6–23 months in North Benin [20]. Baobab fruits and fresh young leaves and seeds are known for their nutritional and pharmacological value and are widely used as food and for medicinal purposes in Africa; moreover, the baobab leaf powder is applied as anti-stress compounds. They are mainly used to treat fatigue, as a boost for insect effect bites, guinea worm symptoms, and external pains even to treat dysentery. The baobab fruit pulp and seeds powder have been used against cases of stomach illness such as dysentery. The extracted baobab seed oil is used for inflamed gums and is useful for diseased teeth [21,22]. According to Sanchez et al. [23], baobab products have been known for many centuries and are widely consumed due to the nutritional deficiencies and low income of African societies. The baobab leaves have been cooked as a soup by the local residence of northern Nigeria [24]. In Malawi, the baobab leaves are boiled with potash which is a plant-based nutrition that contains potassium [25]. In Zimbabwe, rural people are consuming fresh baobab leaves as an alternative for leafy vegetables [26]. In Mali, the leaves are used as a sauce mixed with various indigenous food sources such as okra, onion, meat or fish, and other foodstuff [27].

Although baobab fruit [28], which is approved for consumption in EU countries [29], has attracted greater research interest, baobab leaves are traditionally consumed but no study has integrated and considered them from an interventional point of view.

The main aim of this study was to propose a protocol for a dietary intervention using baobab leaves (BLs) to achieve the recommended reference values for proteins and minerals (K, Ca, Mg, Na, Fe, Mn) for different target groups of the Sudanese population, based on assessing the current health situation and poverty as evidence for the proposed intervention. The specific objective is to determine the nutritional content of BLs such as protein and fat content, minerals (Na, Mg, K, Ca, Fe, Mn), total phenolic and flavonoid compounds, ash content, dry matter, and pH among in the different study areas and to estimate the amount of BLs that should be included in the diet of the target age groups.

## 2. Materials and Methods

### 2.1. Plant Materials

**Figure 1:**
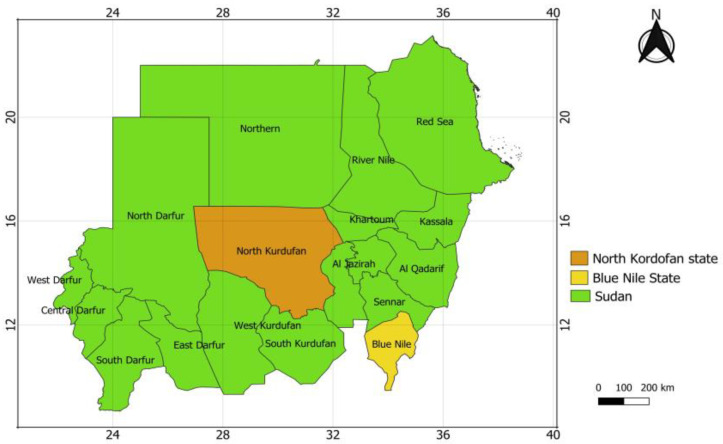
The location of the north Kordofan and Blue Nile regions in Sudan where baobab leaves were sourced. Source, QGIS 3.20.1 software.

**Figure 2:**
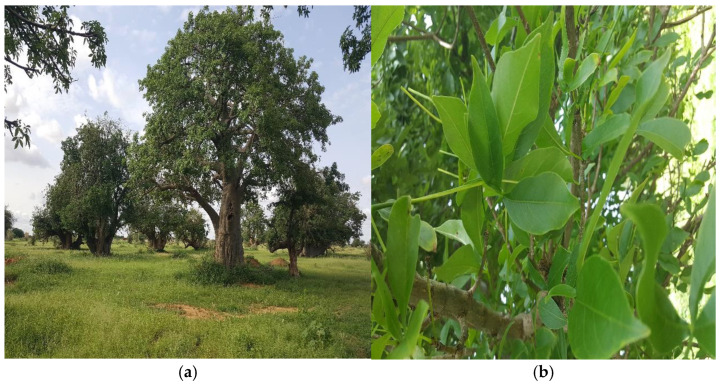
(**a**) Baobab trees and (**b**) baobab of (mixed of young and old) fresh leaves from Sudan.

Baobab trees are mainly distributed in two different regions in Sudan, north Kordofan (KR) and Blue Nile (BN) Figure 1 [30]. In addition, *Adansonia digitata* trees are found in different areas including sloping areas such as the mountains [31]. Based on this, samples of (Mixed of young and old) fresh BLs (Figure 2b) were collected from six areas in both north Kordofan and Blue Nile regions: Khor Tagat (KR1), Kazgil (KR2), Jabal Kordofan (KR3), Er Roseires (BN1), Abu Hashim (BN2), and El Gari (BN3). Ten baobab trees (Figure 2a) were randomly selected in each area and two samples of BLs per tree weighing approximately 3 kg were collected. The collected BLs were dried under a shed at room temperature 25 °C to preserve their nutritional characteristics and then packed in transparent, totally tightened, and sealed polyethylene bags for analysis.

The dried BLs from each site were cleaned of dust material, mixed, ground with an electric grinder, and homogenized. The resulting powder was stored in labeled plastic containers until the analysis was performed in the laboratories of the Institute of Food Science, the University of Debrecen, Hungary.

### 2.2. Mineral Analysis

The content of macro- (Ca, K, Mg, Na) and microelements (Fe, Mn) in the BLs powder was determined by (Thermo Fisher Scientific iCAP 6300, Waltham, MA, USA) inductively coupled plasma atomic emission spectroscopy (ICP-AES) according to the method and protocol for digestion and preparation of calibration standard solutions, concentrated HNO_3_ (65% *w*/*w*, Scharlau Chemie, Spain) and concentrated H_2_O_2_ (30% *w*/*w*, Merck, Germany) solutions were used. To prepare stock solutions, we utilized mono-element standard solutions (mostly 1000 mg dm^−3^) from Merck and BDH, as well as multi-element standard solutions (100 mg dm^−3^) from Spectrascan (Teknolab, Norway), along with high-purity solid reagents manufactured by Reanal (Budapest, Hungary). High-purity water, used for washing and solution preparation, was produced using a two-stage Millipore water purification system (Paris, France). In the first stage, tap water was processed through a MILLI-RO 5 PLUS unit operating on the reverse osmosis principle, yielding water with a conductivity of 0.1–0.2 μS. In the second stage, this water was further purified by a MILLI-Q RG unit equipped with an ion exchange and bacterial filter described in Kovács et al. [32].

### 2.3. Proximate Compositions Have Been Determined as Follows

#### 2.3.1. Dry Matter Determination (ISO 6496:2001)

The dry matter content was determined by drying and homogenizing the BLs in a drying oven (Binder GmbH, Tuttlingen, Germany) at 103 ± 2 °C to a constant weight, ensuring complete removal of water content. The weight difference before and after drying was used to calculate the dry matter as a percentage of the initial sample weight. This method ensures accurate quantification of the total solids current in the sample [33].

#### 2.3.2. Nitrogen and Crude Protein Content (ISO 5983-2:2005)

The nitrogen content in BLs was determined using the Kjeldahl method (Foss Analytical A/S (Hillerød, DK, Denmark), which contains the digestion of the sample with intense (Sulfuric Acid (H_2_SO_4_): Merck KGaA (Darmstadt, HE, Germany) and a catalyst (copper) Sigma-Aldrich (St. Louis, MO, USA) to transform organic nitrogen into ammonium sulfate. The solution was then alkalized, and the ammonia was distilled into a boric acid solution (Thermo Fisher Scientific (Waltham, MA, USA). The ammonia was titrated with a standardized acid (H_2_SO_4_): VWR International (Radnor, PA, USA) to determine the nitrogen content. The crude protein content was calculated by multiplying the nitrogen value by the conversion factor of 6.25, which undertakes a general nitrogen-to-protein ratio [34].

#### 2.3.3. Crude Fat Content (ISO 11085:2015)

The crude fat content in BLs was determined by means of solvent extraction methods, such as Soxhlet extraction (Büchi Labortechnik AG (Flawil, SG, Switzerland). The sample was pre-dried and finely ground to facilitate proficient extraction. An organic solvent (petroleum ether, Merck KGaA (Darmstadt, HE, Germany)) was used to dissolve the fat content. The solvent was vaporized, and the residue (crude fat) was weighed. The fat content was stated as a percentage of the dry sample weight and then converted to g/100 g dry weight [35].

#### 2.3.4. Crude Ash Content (ISO 5984:2002)

The crude ash content of BLs was determined by heating the sample using an oven (Muffle Furnace: Nabertherm GmbH (Lilienthal, NI, Germany) at 550 ± 25 °C until all organic matter was completely combusted. Only the mineral excess (ash) remained, which was cooled in a desiccator (IKA^®^-Werke GmbH & Co. KG, Staufen, BW, Germany) and weighed. The ash content was stated as a percentage of the original sample weight, reflecting the total inorganic mineral content, and calculated as g/100 g dry weight [36].

#### 2.3.5. pH Value Determination (Hungarian Standard MSZ-08-0206-2:1978)

The pH was measured by mixing homogenized the BLs with distilled water (typically in a 1:10 ratio by weight) and allowing the suspension to stabilize. The mixture was stimulated and left to stand for equilibration (Calibration Buffers for pH Meter: Thermo Fisher Scientific Inc. (Waltham, MA, USA), ensuring proper dissolution of pH-active components. A calibrated pH meter (Mettler-Toledo International Inc., Columbus, OH, USA) was used to measure the pH value directly [37].

### 2.4. Determination of Antioxidant Content

Five grams of BLs powder was diluted to 50 mL with a mixture of methanol/water 80:20 (*v*/*v*) and allowed to stand for 6 h, then filtered through folded filter paper (grade 292, Sartorius Stedim Biotech GmbH, Göttingen, Germany). The principle of the method is that phosphotungstic and phosphomolybdic acid found in the Folin–Ciocalteu reagent oxidize phenolic compounds, resulting in a blue-colored solution. Color intensity is proportionate to the concentration of phenolic compounds; therefore, the absorbance of the mixtures is measured using a spectrophotometer (Evolution 300 LC, Thermo Electron Corporation, Oxford, UK) at a wavelength of 760 nm, against the mixture of methanol and distilled water (80:20). To prepare the calibration solutions, a gallic acid stock solution is used. Applied chemicals: 3,4,5-trihydroxybenzoic acid (Alfa Aesar GmbH & Co. KG, Karlsruhe, Germany), sodium carbonate (Sigma-Aldrich Chemie GmbH, Taufkirchen, Germany), methanol (Scharlab S.L., Barcelona, Spain), Folin–Ciocalteu reagent (VWR International S.A.S., Rosny-sous-Bois, France). The results of total phenolic content (TPC) were expressed as mg gallic acid equivalent (GAE)/100 mL [38,39]. The determination of flavonoid content was also carried out by a spectrophotometric method. The absorbance of the rose-colored complex created during the analysis was measured at a wavelength of 510 nm by spectrophotometer (Evolution 300 LC, Thermo Electron Corporation, Oxford, UK) against a blank solution. To prepare the calibration solutions, a catechin stock solution was used. Applied chemicals: catechin (Cayman Chemical Company, Ann Arbor, MI, USA), aluminum chloride (Scharlab S.L., Spain), sodium nitrite (Scharlau Chemie S.A., Barcelona, Spain), sodium hydroxide (Sigma-Aldrich Chemie GmbH, Germany), methanol (Scharlab S.L., Spain). The total flavonoid content (TFC) was expressed as mg catechin equivalent (CE)/100 mL [40,41,42]. Nine replicates were performed for all the above studies.

### 2.5. Estimation of Baobab Leaf Intake (BLI)

The estimated amount of BLs to be included in the diet of the target groups (children 1–3 years, C1; children 4–6 years, C2; children 7–10 years, C3; children 10–18 years, C4; adults ≥ 25 years, AD; pregnant women, PW; lactating women, LW) was calculated as follows:

(1) $$BLI\;\left(g/day\right)=\left(\frac{DRV}{\alpha \times TNB}\right)\times 1000$$

where DRV is the reference value recommended by the European Food Safety Authority for a specific nutrient (Table 1) [43,44,45,46,47,48,49], TNB is the total amount of a specific nutrient in baobab leaves and α is the invitro bioavailability values. According to [50], the values of α for Ca and Mg were 31.5% and 59%, respectively.

To assess the health situation in Sudan, time-series data (2013–2023) from the DataBank database (World Bank Group) Health Nutrition and Population Statistics (https://databank.worldbank.org/source/health-nutrition-and-population-statistics) were used accessed on 10 November 2024. The following indicators were selected: current health expenditure as a percentage of gross domestic product (GDP%), current health expenditure per capita (current USD), out-of-pocket expenditure per capita (current USD), prevalence of anemia, prevalence of hypertension, prevalence of undernourishment, rural population, and urban population.

### 2.6. Statistical Analysis

The data analysis was carried out using the software R (version 4.4.1). Primarily, descriptive statistics (mean and standard deviation) were generated for each nutritional value in the different study areas. The ANOVA test at 5% significant was performed to test for differences in leaf nutrients. The Tukey HSD (Honest Significant Difference) post hoc test was then performed to determine specific differences between groups. Descriptive analyses were performed to test the relationship between health indicator factors.

## 3. Results

### 3.1. Health Condition and Poverty

**Figure 3:**
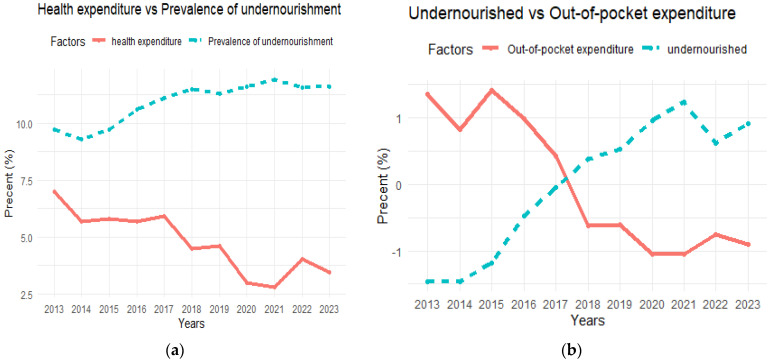
(**a**) Current health expenditure as a percentage of gross domestic product (GDP%) vs. prevalence of undernourishment. (**b**) Out-of-pocket expenditure per capita vs. number of undernourished people.

**Figure 4:**
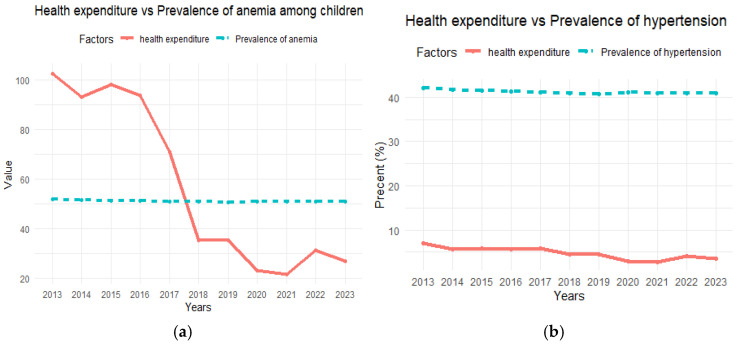
(**a**) Current health expenditure per capita vs. prevalence of anemia among children. (**b**) Current health expenditure as a percentage of gross domestic product (GDP%) vs. prevalence of hypertension among adults.

**Figure 5:**
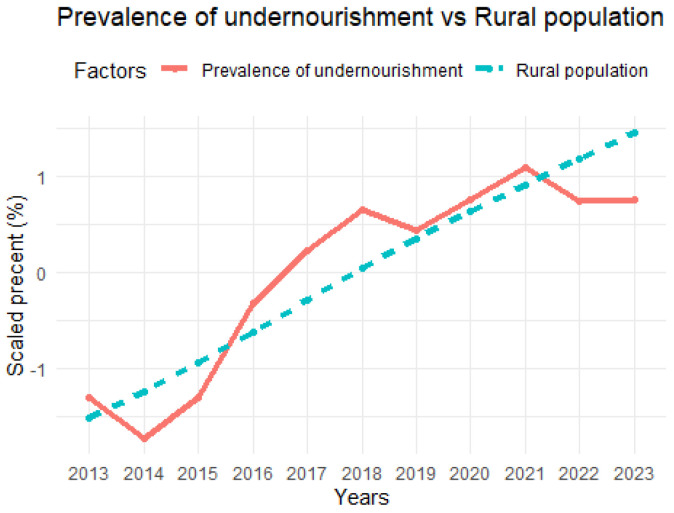
Number of rural population vs. prevalence of undernourishment.

The results of the assessment of health and nutritional status in Sudan are summarized in Figure 3, Figure 4 and Figure 5. Average health expenditure (GDP%) was very low at 2.8%, which is related to a high prevalence of undernourishment among the population. In addition, per capita out-of-pocket expenditure was low at USD72.4 per year, which is linked to the increasing number of undernourished people and indicates limited access to basic health services due to economic constraints (Figure 3). The low annual per capita health expenditure (USD21.6) was associated with a constant prevalence of anemia, which affected a large proportion of the population, especially children, and reproductive and pregnant women (50.8%, 36.3%, and 36.8%, respectively). Despite a decrease in current health expenditure, the prevalence of hypertension remained constant and at a high level of 40.8%, suggesting that poor health care leads to persistent human health issues (Figure 4). The rural population increases by 2.5% annually, this consistent growth rate result makes up a significant percentage of the total population and shows a high increase in the prevalence of undernourishment during the study period (Figure 5). In urban areas, the growing population and high prevalence of undernourishment were also striking, highlighting the link between poverty, lack of access to food, and poor health services.

### 3.2. The Nutritional Levels of Baobab Leaves

The results of the nutrient content of BLs from different areas in Sudan showed great differences (Table 2). Area KR1 had the highest content of Na (86.52 ± 8.12 mg/kg) and the lowest value of Mn (20.37 ± 1.16 mg/kg). The highest concentration of Mg (6263 ± 227 mg/kg), Fe (198 ± 8 mg/kg), Mn (96.30 ± 0.68 mg/kg), and TFC (1554 ± 17 mg/100 g) was found in KR2. The highest crude fat content (2.28 ± 0.02 g/100 g) and the lowest K content (11,218 ± 21 mg/kg) were found in KR3. The lowest Fe level (118 ± 3 mg/kg) and the highest dry matter content (92.72 ± 0.08 g/100 g) were found in BN1. BN2 had the highest crude protein content (16.86 ± 0.01 g/100 g), TPC (396 ± 8 mg/100 g), and the lowest value for dry matter (91.86 ± 0.07 g/100 g), Na (66.40 ± 0.63 mg/kg), Mg (4307 ± 55 mg/kg), and TFC (4307 ± 55 mg/100 g). In addition, BN3 had the highest ash content (12.83 ± 0.01), pH (6.12 ± 0.03), Ca (30,712 ± 940 mg/kg) and K (18,312 ± 286 mg/kg), and the lowest values for crude fat (1.49 ± 0.01 g/100 g), protein (13.99 ± 0.50 g/100 g), and TPC (338 ± 7 mg/100 g). The ANOVA test indicated that the level of all parameters in this study was significantly different (*p* ≤ 0.05) at 5%, except for the dry matter.

**Figure 6:**
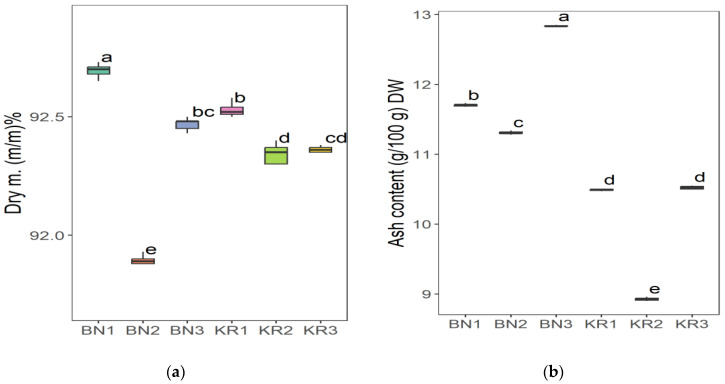
(**a**) Dry matter and (**b**) crude ash content in baobab leaves from six different areas in Sudan. Different letters indicate statistically significant differences (*p* ≤ 0.05). Study areas: Khor Tagat (KR1), Kazgil (KR2), Jabal Kordofan (KR3), Er Roseires (BN1), Abu Hashim (BN2), and El Gari (BN3).

**Figure 7:**
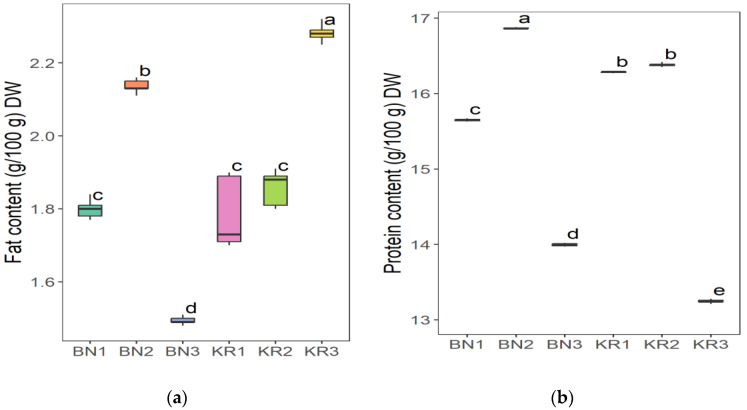
(**a**) Crude fat and (**b**) crude protein content in baobab leaves from six different areas in Sudan. Different letters indicate statistically significant differences (*p* ≤ 0.05). Study areas: Khor Tagat (KR1), Kazgil (KR2), Jabal Kordofan (KR3), Er Roseires (BN1), Abu Hashim (BN2), and El Gari (BN3).

**Figure 8:**
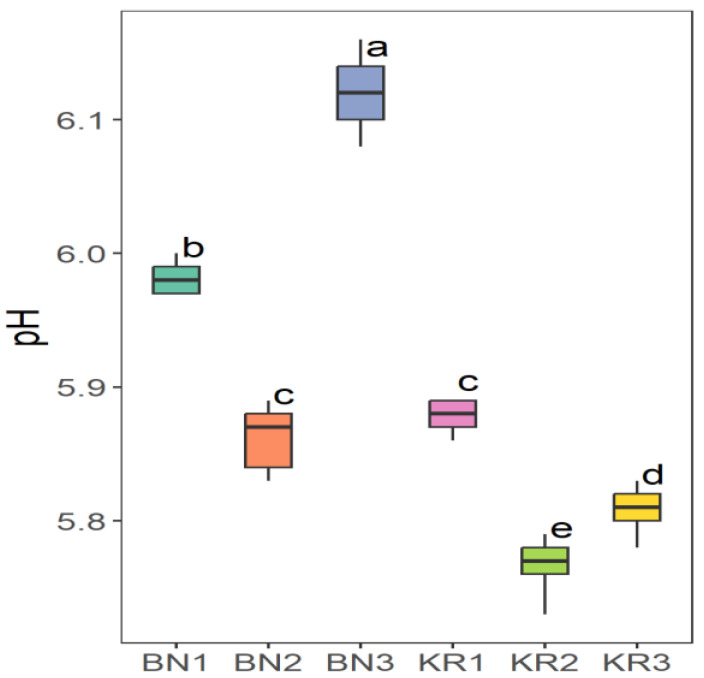
pH values of baobab leaves from six different areas in Sudan. Different letters indicate statistically significant differences (*p* ≤ 0.05). Study areas: Khor Tagat (KR1), Kazgil (KR2), Jabal Kordofan (KR3), Er Roseires (BN1), Abu Hashim (BN2), and El Gari (BN3).

**Figure 9:**
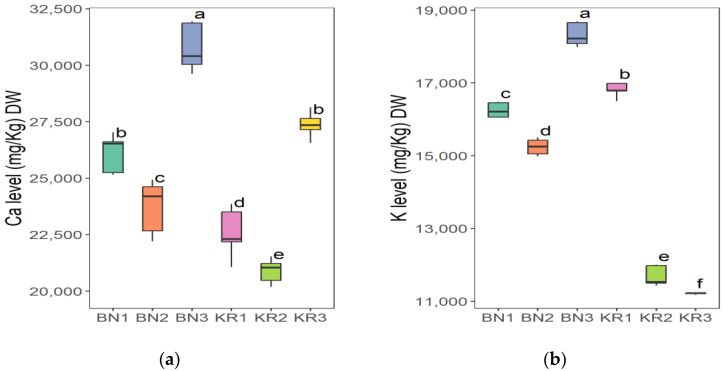
(**a**) Calcium and (**b**) potassium content in baobab leaves from six different areas in Sudan. Different letters indicate statistically significant differences (*p* ≤ 0.05). Study areas: Khor Tagat (KR1), Kazgil (KR2), Jabal Kordofan (KR3), Er Roseires (BN1), Abu Hashim (BN2), and El Gari (BN3).

**Figure 10:**
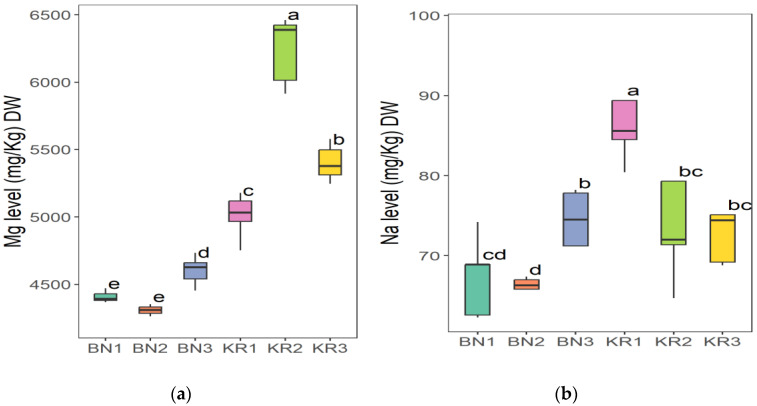
(**a**) Magnesium and (**b**) sodium content in baobab leaves from six different areas in Sudan. Different letters indicate statistically significant differences (*p* ≤ 0.05). Study areas: Khor Tagat (KR1), Kazgil (KR2), Jabal Kordofan (KR3), Er Roseires (BN1), Abu Hashim (BN2), and El Gari (BN3).

**Figure 11:**
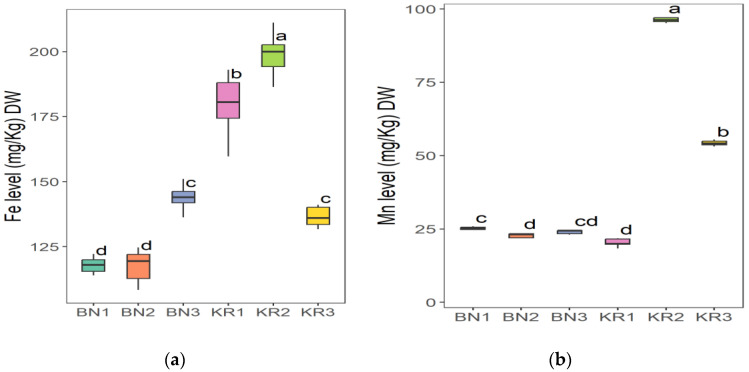
(**a**) Iron and (**b**) manganese content in baobab leaves from six different areas in Sudan. Different letters indicate statistically significant differences (*p* ≤ 0.05). Study areas: Khor Tagat (KR1), Kazgil (KR2), Jabal Kordofan (KR3), Er Roseires (BN1), Abu Hashim (BN2), and El Gari (BN3).

**Figure 12:**
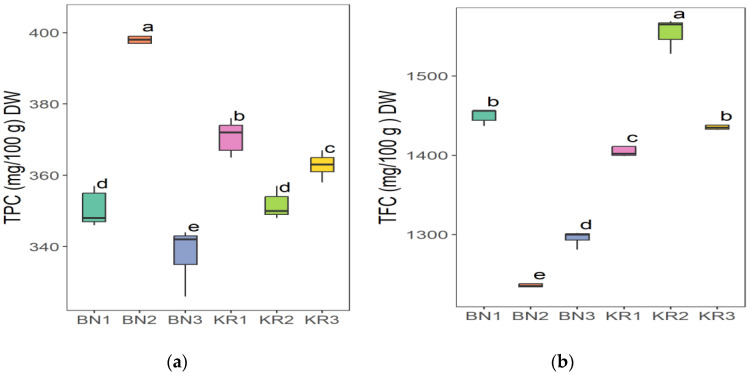
(**a**) TPC (total polyphenols content) and (**b**) TFC (total flavonoid content) in baobab leaves from six different areas in Sudan. Different letters indicate statistically significant differences (*p* ≤ 0.05). Study areas: Khor Tagat (KR1), Kazgil (KR2), Jabal Kordofan (KR3), Er Roseires (BN1), Abu Hashim (BN2), and El Gari (BN3).

The Tukey HSD (Honest Significant Difference) post hoc test was applied to assess the significance of differences between the mean values of nutrient contents of BLs from different Sudanese areas. The results showed that there were no significant differences (ns) between some pairs of the compared study sites: (KR2-KR3) pair for dry matter and (KR1-KR3) for ash content (Figure 6), (BN2-KR1) and (KR1-KRT) for fat content (Figure 7a), and a significant difference between all pairs of mean values for crude protein (Figure 7b). Additionally, (BN2-KR1) pair showed no significant differences for pH (Figure 8). For Ca and K, there was a significant difference between all pairs (Figure 9), while for Mg (Figure 10a), there was also a significant difference. However, for Na, no significant differences were observed between (BN1-BN2), (BN3-KN3), (BN3-KR2), and (KR2-KR3) (Figure 10b). For Fe, no significant differences were noted for the (BN1-BN2) pair (Figure 11a), whereas for Mn, a significant difference was found between all pairs (Figure 11b). Furthermore, the results showed that there was a significant difference between all mean values except between (BN1-KR2) for TPC and (BN1-KR3), (BN1-KR1), and (KR1-KR3) for TFC (Figure 12).

### 3.3. The Estimated Amount of Baobab Leaves (BLI) as a Food Supplement

The calculated amounts of daily intake of baobab leaves (BLI g/day) (children 1–3 years, C1; children 4–6 years, C2; children 7–10 years, C3; children 10–18 years, C4; adults ≥ 25 years, AD; pregnant women, PW; lactating women, LW) are summarized in Table 3, Table 4, Table 5, Table 6, Table 7, Table 8 and Table 9 based on dietary reference values recommended by the European Food Safety Authority in Table 1.

The amount of BLI required to achieve the recommended daily K intake ranged from 44 to 357 g/day in all study areas. The highest intake across all age groups was found for KR3 leaves and the lowest for BN3. C1 had the lowest intake requirement, especially for BN3 leaves. Groups C4, AD, PW, and LW had a uniform BLI in all study areas, ranging from 191 to 357 g/day (Table 3). The amount of BLI to achieve the daily calcium reference intake for the different groups ranged from 47 to 175 g/day. The lowest intake was observed for leaves from area BR3 and the highest for leaves from area KR2. Group C1 required a lower BLI than the other groups (C2–C4, AD, PW, LW) (Table 4). The highest BLIs to cover the daily reference intake of magnesium were found for leaves from the BN2 area and the lowest for leaves from the KR2 area. The AD-M group showed the highest values, particularly for leaves from BN2 (138 g/day). In contrast, C1 had the lowest amounts of BLI, especially for leaves from KR2 (46 g/day). Males required higher BLI than females in groups C4 (81–118 g/day vs. 68–98 g/day) and AD (95–138 g/day vs. 81–118 g/day) (Table 5). The lowest BLI for compliance with the recommended daily sodium reference values was found in all groups for leaves from KR1, while leaves from BN2 had the highest BLIs. For all vegetable materials, the BLIs increased with the age of the target group (Table 6). Women (AD-W, PW and LW groups) required the highest BLI (81–136 g/day) to reach the recommended daily iron intake, especially for BN1 and BN2 leaves. The BLI values for KR2 leaves were the lowest in all sampling areas (Table 7). The dietary recommendations for manganese are limited to the AD, PW, and LW groups, with no differences between them (Table 1). The BLIs were very low, ranging from 0.5 (KR2 area) to 0.7 g/day (BN2 area) (Table 8). The estimated BLI for adequate protein intake ranged widely from (C1–C4 groups) to 144 g/day (LW, first 6 months). The baobab leaves of KR3 were the least effective (highest BLIs), while the BN2 leaves had the lowest BLIs (4–166 g/day) (Table 9).

## 4. Discussion

### 4.1. Current Health Situation

The results of this study show a clear association between low health expenditure and the deterioration of the nutritional and health situation of rural and urban populations in Sudan, which is consistent with the reports of Balla et al. [51] and Ebaidalla et al. [52]. The burden of malnutrition, especially among pregnant and reproductive women and children, highlights the need for serious action as a nutritional intervention as a prevention strategy [53,54].

### 4.2. Nutritional Composition of Baobab Leaves

The study of BLs from different locations shows the nutritious potential of this plant material as a healthy food suitable to meet the nutritional needs of different population groups [55,56]. The parameters studied are discussed below and how these results emphasize their importance.

The crude protein content found in this context is consistent with the results of previous studies [50,57,58]. BLs have a high protein content and can be a reputable source of plant proteins. The protein content is valuable for children and adolescents as it promotes growth and development [59]. It also supports muscle maintenance in adults and the elderly [56,60]. A low crude fat content was found, which is consistent with the fat values of baobab leaves, as reported by other authors [61,62]. The existence of monounsaturated fatty acids could help all age groups, mostly adults and the elderly, by supporting cardiovascular health [62,63]. The dry matter and ash contents agree with the results reported by other researchers [63,64,65]. The high dry matter content in BLs indicates their nutrient density, while the ash content shows their richness in minerals. The mineral content is beneficial for all target groups as it contains macro- and micronutrients that are important for various body functions [66,67,68,69]. The pH of BLs was lower than that reported by Assogbadjo et al. [70], but was within the range of many leafy vegetables [71], suggesting that they are suitable for general consumption without causing acid-related problems.

The evaluation of antioxidants (TPC, TFC) is considered in this study to demonstrate the potential of BLs for human well-being, as phenolic compounds and flavonoids are known for their antioxidant activities, which are crucial for scavenging free radicals and reducing oxidative stress in the body [72,73,74]. The TPC levels found in the BLs of this study were significantly lower than those reported in previous studies, but the TFC levels were similar and comparable to those reported by Babiker et al. [28].

The levels of Ca, K, Mg, and Na found in the BLs in this study are consistent with previous studies [67,75,76]. Macro elements are important for bone well-being, electrolyte stability, and muscle function [77,78]. In particular, the K content of BLs can support blood pressure regulation [79], while high Mg and Ca content supports bone development in children and adolescents and helps prevent bone-related problems [80]. BLs are also rich in the microelements Mn and Fe [66,76], which is consistent with the results of our study. Iron supports the prevention of dangerous diseases such as anemia, especially in children, pregnant women, and women in the reproductive phase [81,82]. Mn content is helpful for metabolism, especially in adults and adolescents who exercise regularly [83,84]. Referring to our study results and previous research findings, we found that BLs are reliably emphasized as a nutrient-rich food source. Nevertheless, this study proves that BLs from different areas may have slight variations in nutrient content due to the adaptability of baobabs to ecological factors such as soil type and macroclimate, which may affect the estimated BLI of different sites [85].

### 4.3. Key Implications of the Research

The BLs is a healthy dietary ingredient that supports strength at different ages. Their high total content of phenols, flavonoids, minerals, and proteins makes them beneficial for the human body. This study shows that BLs have significant potential as a healthy food that can meet the nutritional needs of different people. Thus, the systematic inclusion of BLs in the diet can positively support the nutritional status of children, adults, and pregnant and lactating women.

Therefore, the BLI (g/day) which is calculated using the recommended daily nutrient intake for each target group is strongly recommended to be included, as a healthy diet to combat malnutrition. The estimated amount of baobab leaves powder should be added to the available cooked sauce or soup (i.e., onion, tomato, salt, etc.) commonly consumed by the local communities as an alternative to economical leafy vegetables in each specific area.

**Figure 13:**
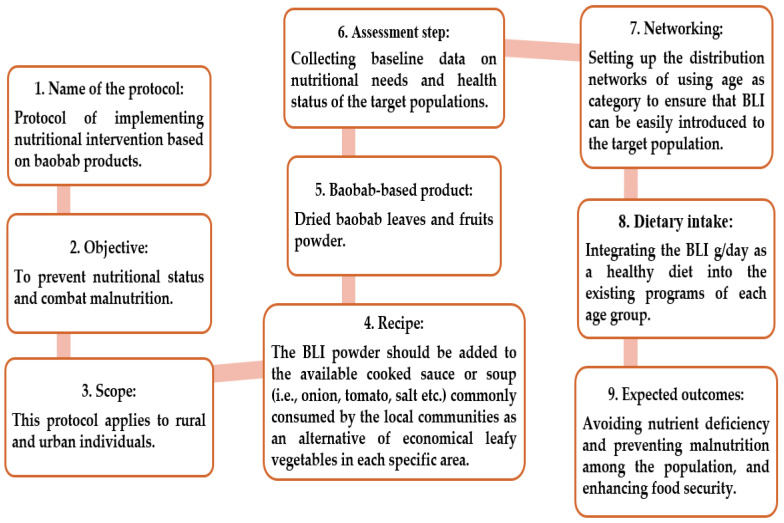
Protocol steps for implementing nutritional intervention using baobab leaves (BLs).

The study area with the lowest amount of BLs to be consumed is recommended. Leaves from the area of El Gari (BN3) 18.312 g/day and 30.712 g/day are recommended for K and Ca particularly beneficial for children aged 1–3 years and lactating women. Leaves from Kor Tagat (KR1) are suitable for sodium intake, requiring approximately 13–23 g/day across all age groups. Leaves from Kazgil (KR2) (46–81 g/day), (35–66 g/day), (0.48–0.68 g/day), and (4–6 g/day) are optimal for fulfilling the daily requirements of magnesium, iron, manganese, and protein in this order. These findings can be used to advance the dietary intervention protocol based on the consumption of BLs, as shown in Figure 13.

## 5. Strengths and Limitations

This study climaxes the nutritional potential of BLs as a dietary intervention by proposing a scientific protocol to address nutrient deficiencies and poverty-related health issues. It offers an inclusive nutritional analysis using internationally known standard methods, ensuring reliability and precision. The inclusion of samples from diverse regions across Sudan improves the applicability of the findings. Daily dietary recommendations for varied demographic groups, such as children, adults, and pregnant women, reveal the likely public health influence of baobab products in resource-limited situations. Additionally, the study lays a strong basis for future research into the broader applications and health assistance of baobab-based diet.

Nevertheless, some limitations are necessary to be accredited. The study did not evaluate the bioavailability of nutrients, which could affect their efficiency in human diets. It also required clinical trials or community-level interventions to approve the health benefits. Moreover, anti-nutritional aspects that could upset nutrient absorption were not examined. Lastly, experiments related to large-scale farming, collecting, and treating to support wider population needs were not addressed.

## 6. Recommendation

To confirm the safety of BLI for consumption, future research should focus on the following: (i) assessing the content of potentially toxic elements such as Pb, Cd, As, and Cr in BLs powder, especially considering long-term health risks; (ii) investigating the load of pathogens such as *Salmonella* spp. and *Escherichia coli* to confirm the microbiological well-being of the leaves; (iii) assessing the nutritional composition factors that could affect the absorption of nutrients such as phytates and oxalates; (iv) investigating the in vitro bioavailability (i.e., α) of various elements such as K, Mn, Na, and Fe. Target Hazard Quotient (THQ) should be assessed and calculated for non-carcinogenic health hazards and Incremental Lifetime Cancer Risk (ILCR) should be used to quantify cancer risk for various chemical contaminants. These recommended areas of research will help to certify the safety of BLs for use in dietary interventions.

In addition, further research should be carried out to improve the availability of Fe and increase its absorption for different target groups. Fermentation of BLs can reduce antinutrients that impair Fe absorption [86,87,88]. The addition of food supplements such as ferrous gluconate and ferrous sulfate can improve the Fe content of BLs to ensure the recommended intake of new processing techniques and different cooking methods (e.g., boiling and steaming) should also be explored to improve the bioavailability of Fe and reduce the content of antinutritional factors [89,90].

## 7. Conclusions

The study results highlight the need for health policies that will tackle malnutrition and food insecurity by incorporating naturally available, nutritious alternatives like the BLs. Policy-makers worldwide should consider and integrate the proposed protocol into public health nutritional programs, specifically in underserved and rural areas, to alleviate the micronutrient deficiencies and their consequences for health problems, such as undernourishment, anemia, and hypertension. Investing in food security and rural development initiatives will decrease disparities due to low health expenditure and economic hurdles. In the context of clinical practice, the study points out the potential for combining nutrient-dense plant-based choices into dietary guidelines. Health care providers should combat malnutrition-related diseases by advocating for culturally acceptable and feasible interventions. Screening programs for vulnerable populations must be a priority and adoption of dietary counseling to include locally available food solutions.

## Figures and Tables

**Table 1:** Dietary reference values recommended by the European Food Safety Authority.

Group/Age	Potassium (mg/Day)	Calcium (mg/Day)	Magnesium (mg/Day)	Sodium (g/Day)	Protein (g/kg/Day)	Iron (mg/Day)	Manganese (mg/Day)
Children (1–3 years)	800	450	170	1.1	0.66	7	-
Children (4–6 years)	-	800	230	1.3	0.66	7	-
Children (7–10 years)	-	800	230	1.7	0.66	11	-
Children (10–18 years)	3500	1150	B: 300, G: 250	2.0	0.66	B: 11, G: 13	-
Adults (≥25 years)	3500	750	M: 350, W: 300	2.0	0.83	M: 11, W: 16	3
Pregnant Women	3500	750	300	2.0	1 g (1st), 9 g (2nd), 28 g (3rd)	16	3
Lactating Women	4000	750	300	2.0	19 g (FI), 13 g (AF)	16	3

1st trimester, 2nd trimester, 3rd trimester. “B”: Boys, “G”: Girls, “M”: Men, “W”: Women, “-” value is not available or not applicable. “FI”: (First 6 months), “AF”: (After 6 months). Source: [43,49].

**Table 2:** Nutritional levels of baobab leaves (means ± SD, *n* = 9).

	KR1	KR2	KR3	BN1	BN2	BN3
Dry matter (m/m%)	92.6 ^a^ ± 0.028	92.3 ^a^ ± 0.089	92.4 ^a^ ± 0.094	92.7 ^a^ ± 0.075	91.8 ^a^ ± 0.072	92.45 ^a^ ± 0.026
Ash (g/100 g)	10.49 ^a^ ± 0.051	8.93 ^b^ ± 0.02	10.53 ^c^ ± 0.127	11.69 ^d^ ± 0.035	11.30 ^e^ ± 0.021	12.83 ^f^ ± 0.01
Fat (g/100 g)	1.79 ^a^ ±0.093	1.86 ^b^ ± 0.045	2.28 ^c^ ± 0.021	1.79 ^d^ ± 0.041	2.14 ^e^ ± 0.016	1.49 ^f^ ± 0.013
Protein (g/100 g)	16.29 ^a^ ± 0.012	16.38 ^b^ ± 0.021	13.24 ^c^ ± 0.025	15.65 ^d^ ± 0.012	16.86 ^e^ ± 0.014	13.99 ^f^ ± 0.5
pH	5.88 ^a^ ± 0.012	5.76 ^b^ ± 0.021	5.81 ^c^ ± 0.016	5.97 ^d^ ± 0.036	5.86 ^e^ ± 0.024	6.12 ^f^ ± 0.026
Ca (mg/kg)	22,342 ^a^ ± 1292	20,917 ^b^ ± 480	27,364 ^c^ ± 498	26,177 ^d^ ± 748	23,803 ^e^ ± 1076	30,712 ^f^ ± 940
K (mg/kg)	16,826 ^a^ ± 163	11,662 ^b^ ± 250	11,218 ^c^ ± 21.47	16,243 ^d^ ± 183	15,244 ^e^ ± 196	18,312 ^f^ ± 286
Na (mg/kg)	86.52 ^a^ ± 8.12	73.11 ^b^ ± 5.23	72.91 ^c^ ± 2.95	67.021 ^d^ ± 4.03	66.4 ^e^ ± 0.63	74.56 ^f^ ±3.11
Mg (mg/kg)	5015 ^a^ ± 133	6263 ^b^ ± 227	5394 ^c^ ± 120	4413 ^d^ ± 49.985	4307 ^e^ ± 55	4604 ^f^ ± 89.9
Fe (mg/kg)	179 ^a^ ± 10.06	198 ^b^ ± 7.91	136.5 ^c^ ± 3.68	117.9 ^d^ ± 2.92	118 ^e^ ± 5.85	144.6 ^f^ ± 5.61
Mn (mg/kg)	20.37 ^a^ ± 1.16	96.30 ^b^ ± 0.68	54.20 ^c^ ± 0.85	25.29 ^d^ ± 0.434	20.6 ^e^ ± 6.69	24.07 ^f^ ± 0.65
TPC (mg/100 g)	371 ^a^ ± 4.26	351 ^b^ ± 3.31	363 ^c^ ± 3.06	351 ^d^ ± 4.583	396 ^e^ ± 8.36	338 ^f^ ± 7.21
TFC (mg/100 g)	1417 ^a^ ± 28.65	1554 ^b^ ± 16.93	1441 ^c^ ± 13.5	1449 ^d^ ± 8.50	1238 ^e^ ± 12	1294 ^f^ ± 8.63

Study area: Khor tagat (KR1), Kazgil (KR2), Jabal Kordofan (KR3), Er Roseires (BN1), Abu Hashim (BN2), and El Gari (BN3). Different letters on values indicate a significant difference (*p* ≤ 0.05) at 5%.

**Table 3:** The estimated amount of baobab leaf intake (BLI g/day) to meet the daily potassium reference value. The BLI g/day was calculated based on their nutritional means in Table 2, and the dietary reference values in Table 1.

	C1	C4	A	PW	LW
KR1	48	208	208	208	238
KR2	69	300	300	300	343
KR3	71	312	312	312	357
BN1	49	216	216	215	246
BN2	53	229	229	229	262
BN3	44	191	191	191	218

Age groups: children (1–3 years) (C1), children (10–18 years) (C4), adults (≥25 years) (AD), pregnant women (PW), and lactating women (LW). Study area: Khor tagat (KR1), Kazgil (KR2), Jabal Kordofan (KR3), Er Roseires (BN1), Abu Hashim (BN2), and El Gari (BN3).

**Table 4:** The estimated amount of baobab leaf intake (BLI g/day) to meet the daily calcium reference. The BLI g/day was calculated based on their nutritional means in Table 2, and the dietary reference values in Table 1.

	C1	C2	C3	C4	AD	PW	LW
KR1	64	114	114	163	107	107	107
KR2	68	121	121	175	114	114	114
KR3	52	93	93	133	87	87	87
BN1	55	97	97	139	91	91	91
BN2	60	107	107	153	100	100	100
BN3	47	83	83	119	78	77.52	78

Age groups: children (1–3 years) (C1), children (4–6 years) (C2), children (7–10 years) (C3), children (10–18 years) (C4), adults (≥25 years) (AD), pregnant women (PW), lactating women (LW). Study area: Khor tagat (KR1), Kazgil (KR2), Jabal Kordofan (KR3), Er Roseires (BN1), Abu Hashim (BN2), and El Gari (BN3).

**Table 5:** The estimated amount of baobab leaf intake (BLI g/day) to meet the daily magnesium reference. The BLI g/day was calculated based on their nutritional means in Table 2, and the dietary reference values in Table 1.

	C1	C2	C3	C4-B	C4-G	AD-M	AD-W	PW	LW
KR1	57	78	78	101	84	118	101	101	101.
KR2	46	63	62	81	68	95	81	81	81
KR3	53	72	72.	94	78	110	94	94	94
BN1	65	88	88	115	96	96	115	115	115
BN2	67	91	91	118	98	138	118	118	118
BN3	63	85	85	110	92	129	110	110	110

Age groups: children (1–3 years) (C1), children (4–6 years) (C2), children (7–10 years) (C3), children (10–18 years) (C4), adults (≥25 years) (AD), pregnant women (PW), lactating women (LW). Study area: Khor tagat (KR1), Kazgil (KR2), Jabal Kordofan (KR3), Er Roseires (BN1), Abu Hashim (BN2), and El Gari (BN3).

**Table 6:** The estimated amount of baobab leaf intake (BLI g/day) to meet the daily sodium reference. The BLI g/day was calculated based on their nutritional means in Table 2, and the dietary reference values in Table 1.

	C1	C2	C3	C4	AD	PW	LW
KR1	13	15	20	23	23	23	23
KR2	15	18	23	27	27	27	27
KR3	15	18	23	27	27	27	27
BN1	16	19	25	30	30	30	30
BN2	17	20	26	30	30	30	30
BN3	15	17	23	27	27	27	30

Age groups: children (1–3 years) (C1), children (4–6 years) (C2), children (7–10 years) (C3), children (10–18 years) (C4), adults (≥25 years) (AD), pregnant women (PW), lactating women (LW). Study area: Khor tagat (KR1), Kazgil (KR2), Jabal Kordofan (KR3), Er Roseires (BN1), Abu Hashim (BN2), and El Gari (BN3).

**Table 7:** The estimated amount of baobab leaf intake (BLI g/day) to meet the daily iron reference. The BLI g/day was calculated based on their nutritional means in Table 2, and the dietary reference values in Table 1.

Age Group	C1	C2	C3	C4-B	C4-G	AD-M	AD-W	PW	LW
KR1	39	39	61	61	73	61	89	89	89
KR2	35	35	56	56	66	56	81	81	81
KR3	51	51	81	81	95	81	117	117	117
BN1	59	59	93	93	110	93	136	136	136
BN2	59	59	93	93	110	93	136	136	136
BN3	48	48	76	76	90	76	111	111	111

Age groups: children (1–3 years) (C1), children (4–6 years) (C2), children (7–10 years) (C3), children (10–18 years) (C4), adults (≥25 years) (AD), pregnant women (PW), lactating women (LW). “B”: Boys, “G”: Girls, “M”: Men, “W”: Women. Study area: Khor tagat (KR1), Kazgil (KR2), Jabal Kordofan (KR3), Er Roseires (BN1), Abu Hashim (BN2), and El Gari (BN3).

**Table 8:** The estimated amount of baobab leaf intake (BLI g/day) to meet the daily manganese reference. The BLI g/day was calculated based on their nutritional means in Table 2, and the dietary reference values in Table 1.

	AD	PW	LW
KR1	0.598	0.598	0.598
KR2	0.479	0.479	0.479
KR3	0.556	0.556	0.556
BN1	0.68	0.68	0.68
BN2	0.697	0.697	0.697
BN3	0.652	0.652	0.652

Age groups: adults (≥25 years) (AD), pregnant women (PW), lactating women (LW). Study area: Khor tagat (KR1), Kazgil (KR2), Jabal Kordofan (KR3), Er Roseires (BN1), Abu Hashim (BN2), and El Gari (BN3).

**Table 9:** The estimated amount of baobab leaf intake (BLI g/day) to meet the daily protein reference. The BLI g/day was calculated based on their nutritional means in Table 2, and the dietary reference values in Table 1.

	C1	C2	C3	C4	AD	PW (1st)	PW (2nd)	PW (3rd)	LW (FI)	LW (AF)
KR1	4.052	4.052	4.052	4.052	5.095	6	55	172	117	79.8
KR2	4.029	4.029	4.029	4.029	5.067	6	55	170	115.9	79.4
KR3	4.985	4.985	4.985	4.985	6.269	7.5	67.9	211.5	143.5	98
BN1	4.217	4.217	4.217	4.217	5.304	6.4	57.5	178.9	121.4	83
BN2	3.915	3.915	3.915	3.915	4.923	5.9	53.3	166	113	77
BN3	4.718	4.718	4.718	4.718	5.933	7	64.3	200	1135	92.9

Age groups: children (1–3 years) (C1), children (4–6 years) (C2), children (7–10 years) (C3), children (10–18 years) (C4), adults (≥25 years) (AD), pregnant women (PW), lactating women (LW). First trimester, second trimester, and third trimester. “B”: Boys, “G”: Girls, “M”: Men, “W”: Women, “FI”: (first 6 months), “AF”: (after 6 months). Study area: Khor tagat (KR1), Kazgil (KR2), Jabal Kordofan (KR3), Er Roseires (BN1), Abu Hashim (BN2), and El Gari (BN3).

## Data Availability

The original contributions presented in the study are included in the article, further inquiries can be directed to the corresponding author.
